# Survival and Nutritional Status of Male and Female Heart Transplant Patients Based on the Nutritional Risk Index

**DOI:** 10.3390/nu12123868

**Published:** 2020-12-17

**Authors:** Deema A. Almutawa, May Almuammar, Mona Mohamed Elshafie, Ghadeer S. Aljuraiban, Alaa Alnafisah, Mahmoud M. A. Abulmeaty

**Affiliations:** 1 Department of Community Health Sciences, College of Applied Medical Sciences, King Saud University, Riyadh 11362, Saudi Arabia; deema.almutawa@gmail.com (D.A.A.); malmuammar@ksu.edu.sa (M.A.); dr.melshafie22@gmail.com (M.M.E.); galjuraiban@ksu.edu.sa (G.S.A.); 2 Health Sciences Department, Princess Nourah Bint Abdulrahman University, Riyadh 11564, Saudi Arabia; 3 Al Ghad International College of Applied Medical Sciences, Riyadh 12467, Saudi Arabia; 4 Clinical Nutrition Department, King Faisal Specialist Hospital and Research Center, Riyadh 12713, Saudi Arabia; AAlnafisah@kfshrc.edu.sa; 5 Obesity Management and Research Unit, Medical Physiology Department, Faculty of Medicine, Zagazig University, Zagazig 44519, Egypt

**Keywords:** malnutrition, gender difference, nutritional risk index, heart transplant, survival

## Abstract

Malnutrition among heart-transplant patients may affect survival. The aim was to investigate the survival and nutrition status among male and female heart transplant patients who underwent transplantation, before and 1 year after surgery based on the nutritional risk index (NRI). The medical records of ninety heart-transplant patients (2009–2014) from the King Faisal Specialist Hospital, Riyadh, were reviewed. The assessment included demographic data, anthropometric measurements, and NRI calculation. Moreover, postoperative data included the length of stay and survival. Paired *t*-test and survival analysis by Kaplan–Meier (KM) curves were used. A total of 90 patients (males 77.78%) were included. The prevalence of malnutrition in the preoperative phase by NRI was 60% (7.78% as severe; 40% as moderate, and 12.22% mild NRI scores). After 1 year, body mass index (BMI) and NRI increased significantly (*p* < 0.001). Furthermore, NRI was significantly different between men and women (*p* < 0.01), while KM survival curves were insignificantly different (*p* = 0.67). Recipients with postoperative moderate or severe nutritional risk (NRI < 97.5) had significantly shorter survival in the first-year post-transplantation (HR = 0.82; 95% CI, 0.75–0.89; *p* < 0.001). Our findings indicate that the NRI after 1 year of transplant correlated significantly with mortality. Besides, there was no significant gender difference regarding survival; however, malnutrition and low survival were more prominent among women.

## 1. Introduction

Nutritional status and heart failure have a strong relationship. The prevalence of heart failure-associated malnutrition was estimated to be up to 70%, and 15% to 50% of patients with heart failure were cachectic globally [1,2,3,4]. However, there are no specific data about the prevalence of malnutrition among heart failure patients living in Saudi Arabia. In the case of severe end-stage heart failure, a heart transplant becomes a life-saving measure. Moreover, the nutrition care process (NCP) is extremely important in the management of patients undergoing transplantation. During all times of transplantation, the nutritional assessment is the cornerstone of NCP which including history, clinical examination, anthropometric measurements, biochemical parameters, and probably some other sophisticated tests such as dual-energy X-ray absorptiometry (DEXA) [4,5,6].

Till now, there have been a variety of nutritional assessment tools; there is no consensus on the best method to assess the nutritional status of pre-transplant heart failure patients. Nevertheless, the nutritional risk index (NRI) was developed and proved to be correlated with increased mortality and morbidity of postoperative patients [7]. Interestingly, the length of hospital stay, the readmission rates, and all-cause mortality were significantly higher in the patients with lower NRI scores [8,9]. Furthermore, it was reported that body mass index (BMI) can be a reliable predictor of post-transplant mortality and morbidity. Heart transplant candidates achieved good post-transplant outcomes on a long-term basis when they were normal weighted, over-weighted, or with class I obesity [10]. Another opinion suggested that patients with BMI equal to or less than 24 kg/m^2^ showed an increased risk of in-hospital and long-term mortality; besides, those with BMI > 35 kg/m^2^ had higher morbidities and postoperative hospital resource use [11]. Surprisingly, many observational studies suggest a protective effect of obesity, which has been known as the obesity paradox. It is plausible that patients with obesity have a more metabolic reserve to overcome the increased catabolic stress that results from major operations [12,13,14,15].

It was proven that gender greatly affects the pathophysiology and clinical manifestation of cardiovascular diseases (CVD) [16]. However, the impact of gender on cardiac transplant-related nutritional deterioration and its prognosis is still under investigation. Hence, we were aiming to evaluate the survival and nutrition status among male and female heart transplant patients who underwent transplantation, before and 1 year after surgery based on the NRI.

## 2. Materials and Methods 

### 2.1. Study Design and Setting

A retrospective cohort study was conducted at King Faisal Specialist Hospital & Research Centre (KFSH&RC) in Riyadh, Saudi Arabia. The medical records of all heart transplant patients from the beginning of 2009 till September 2014, with a 1-year follow-up, were reviewed. The study protocol was approved by the Institutional Review Board of the College of Applied Medical Sciences, King Saud University (ref. No. CAMS 93-36/37), and KFSH&RC project No. 2161051.

### 2.2. Study Sample

Ninety consecutive heart transplant patients with different pre-transplant diagnoses were enrolled in this study. Completed case report forms were designed to report details of past medical diagnosis, demographics, and anthropometric measures. We excluded all transplant patients below the age of 18 years.

### 2.3. Sample Size

The sample size was calculated by G*Power software 3.1.9.4 (University of Kiel, Kiel, Germany), considering alpha error probability at 0.05, power (1-β error probability) equal 0.95, and the effect size d = 0.5. The estimated sample size for the dependent sample *t*-test, befor and after transplantation, was estimated to be 54 participants.

### 2.4. Study Tools

#### 2.4.1. Anthropometric Measurements

Two variables were selected: weight (kg) and height (cm) for calculating body mass index (BMI). Weight and height were measured to the nearest 0.1 kg. Weight was taken by (Scale-Tronix scale, White Plains, NY, USA) and height was taken by a stadiometer (Seca Co, Hamburg, Germany). The participants were divided into 4 groups based on BMI as follows: BMI < 18.5 (underweight), 18.5 to 24.99 (normal weight), 25 to 29.99 (overweight), and ≥30 (obesity).

#### 2.4.2. Malnutrition Assessment

Malnutrition assessment was based on the NRI, which was originally derived from serum albumin concentration and the ratio of the present to the usual weight. However, ideal body weight (IBW) was used instead of the usual body weight (UBW) because of the difficulty in identifying UBW in pre-transplant heart failure patients and IBW is less subjective. Hence, the NRI formula we used was follows: NRI = (1.519 × serum albumin, g/L) + 41.7 × (actual weight, kg/ideal weight, kg) [17]. The IBW was calculated using the Devine formula for men as follows: IBW [kilograms] = 50 kg + 2.3 kg for each inch above 5 feet [18]. Since it is more accurate, the Robinson formula was used for women as follows: IBW [kilograms] = 48.67 kg + 1.65 kg for each inch above 5 feet [19]. When (actual weight, kg/ideal weight, kg) ≥1, then the ratio was set to 1. According to NRI values, four categories of malnutrition were identified. An NRI ≥ 100 suggests no evidence of malnutrition; 97.5 to 100 suggests mild malnutrition; 83.5 to 97.5 indicates moderate malnutrition, and <83.5 indicates severe malnutrition [17].

#### 2.4.3. The Length of Stay

Hospital length of stay means the actual number of days the patients remained at the hospital after heart transplant surgery.

#### 2.4.4. Blood Biochemical Tests

The following tests were collected from electronic files; (a) Complete Blood Count with Differential count (CBCD) such as: hemoglobin (g/L), hematocrit (%), total lymphocyte count (%), and count of red blood cells (1012/L). (b) Iron profile: iron (µmol/L), ferritin (µg/L), total iron-binding capacity (TIBC) (µmol/L), and unsaturated iron-binding capacity (UIBC) (µmol/L). (c) Hepatic profile; total protein (g/dL), albumin (g/L), prealbumin (mg/dL), and alkaline phosphatase (ALP) (U/L). (d) Lipid profile; serum cholesterol (mmol/L), low-density lipoprotein (LDL) cholesterol (mmol/L), high-density lipoprotein (HDL) cholesterol (mmol/L), and Triglycerides (mmol/L). (e) Hormones: triiodothyronine (T3) (mmol/L), free thyroxine (FT4) (Pmol/L), and thyroid-stimulating hormone (TSH) (mU/L).

### 2.5. Statistical Analysis

Data were processed and analyzed using the Statistical Analysis System (SAS) software program version 9.4 (SAS Institute, Cary, NC, USA). Continuous data were expressed as means ± SD, 95% CI. For gender difference, Student’s independent *t*-test was used. Furthermore, the comparison between continuous variables before and one year after transplantation was done using the paired-samples Student’s *t*-test. To study postoperative survival, Kaplan–Meier analysis was used. Cox proportional hazards regression was also performed with 95% CIs. Results were considered statistically significant at *p* < 0.05.

## 3. Results

### 3.1. Participants’ Basal Characteristics

The general characteristics of the study population were shown in Table 1. Males were much greater than females (77.78%). The mean age was about 39.84 ± 12.22 years for males and 32.35 ± 9.31 for females, the mean length of stay was 28.80 ± 26.27 days, and non-significant differences for gender were *p* = 0.5. In the first year post-transplant, 16.67% of the participants died. The diagnoses of pre-transplant conditions were: dilated cardiomyopathy (DCM) (52.22%), ischemic cardiomyopathy (ICM) (32.22%), rheumatoid heart disease (6.67%), restrictive cardiomyopathy, congenital heart disease and hypertrophic cardiomyopathy (2.22%), and chemo-induced cardiomyopathy and postpartum cardiomyopathy (1.11%).

### 3.2. Anthropometric and NRI Comparison Pre- and Post-Transplantation

Table 2 compares the characteristics of heart recipients before and 1 year after transplant. After transplantation, there was a highly significant increase in weight, BMI, and serum albumin levels (*p* < 0.001). As a result, the NRI increased significantly after transplantation (96.60 ± 8.50 vs. 105.50 ± 8.10; *p* < 0.001).

### 3.3. Comparison between Pre-Transplant and Post-Transplant Nutritional Assessment According to Gender

**Figure 1:**
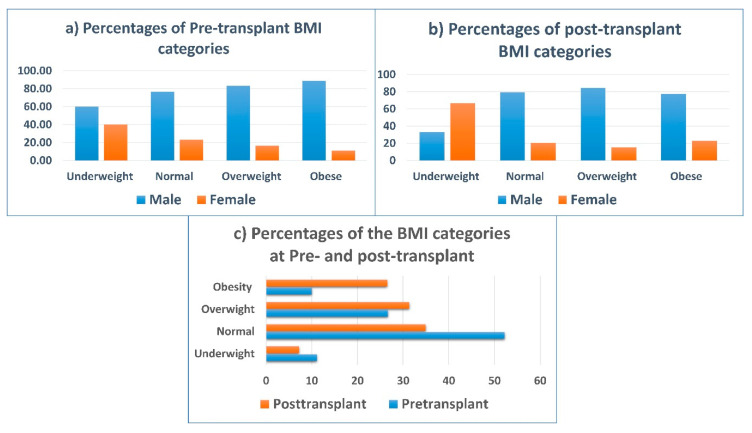
(**a**–**c**): Percentages of the changes in body mass index (BMI) classifications from pre-transplant to 1-year post-transplant according to gender.

Figure 1a–c illustrates percentages of the changes in BMI categories pre- and post-transplantation, pre-transplant underweight, and the normal BMI categories among the female group were more than those of the male one (20% vs. 8.57% and 55% vs. 51.43%, respectively), while the overweight and obesity categories among male patients were more than female patients (28.57% vs. 20% and 11.43% vs. 5%, respectively), (*p* = 0.04). Similarly, after transplantation, the prevalence of underweight BMI remained higher among females than males (21.05% vs. 3.13%) and overweight and obesity prevalence continued higher among males than females (34.38% vs. 21.05% and 26.56% vs. 26.32%); for both BMI baseline and after 1 year, no significant difference was noted in BMI (*p* = 0.18). Moreover, BMI means that pre-transplant for the total population decreased significantly (*p* < 0.001) for post-transplant.

The percentage of overweight and obese patients (BMI ≥ 25 kg/m^2^) increased after 1 year from 36.67% to 57.84%. As a result, the prevalence of normal and underweight patients decreased after 1 year of a transplant from 52.22% to 34.94% and 11.11% to 7.23%, respectively.

Table 3 shows biochemical nutritional assessment parameters between male and female groups before and 1 year after heart transplantation. Hemoglobin concentration, hematocrit value, and red blood cells showed a gender difference in both times of assessment. Interestingly, prealbumin level was also significantly higher in males than females in pretransplant and 1-year post-transplant. The total protein level was significantly higher in men in the post-transplant phase.

**Figure 2:**
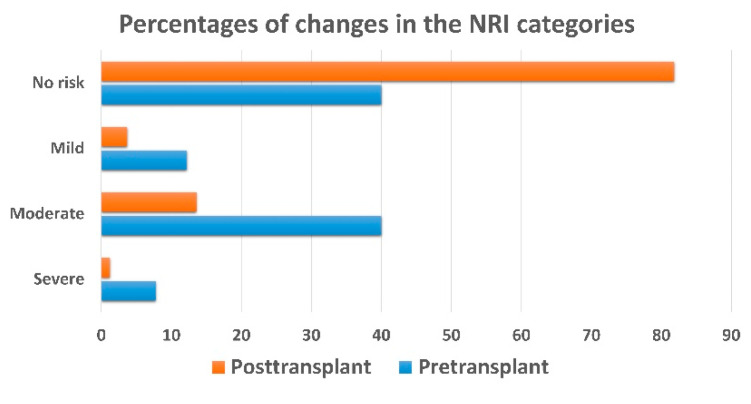
Changes in NRI categories from pre-transplant to 1-year post-transplant.

Regarding NRI Classifications, Figure 2 shows that the prevalence of nutritional risk, by all degrees, was greater at the pre-transplant phase than the post-transplant phase (60% vs. 18.51%). Severe nutritional risk decreased considerably after heart transplant surgery (7.78% to 1.23%). Additionally, the moderate nutritional risk was the most prevalent category before transplantation, and it did not alter after transplantation.

### 3.4. Survival Analysis

**Figure 3:**
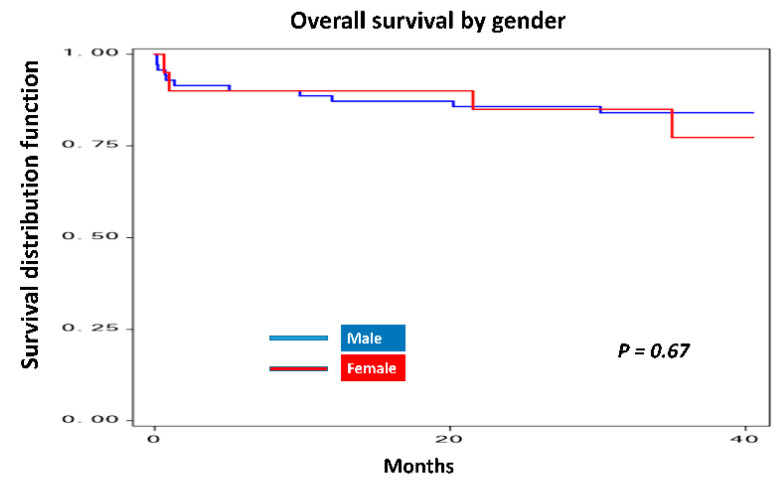
Kaplan–Meier plot of overall survival by gender.

Overall, Kaplan–Meier survival curves by gender (Figure 3) revealed that the males had a slightly higher survival of 84.29% (the mean survival time = 26.6 months), compared to females who had a percentage of survival of 80% (the mean survival time = 30.9 months).

**Figure 4:**
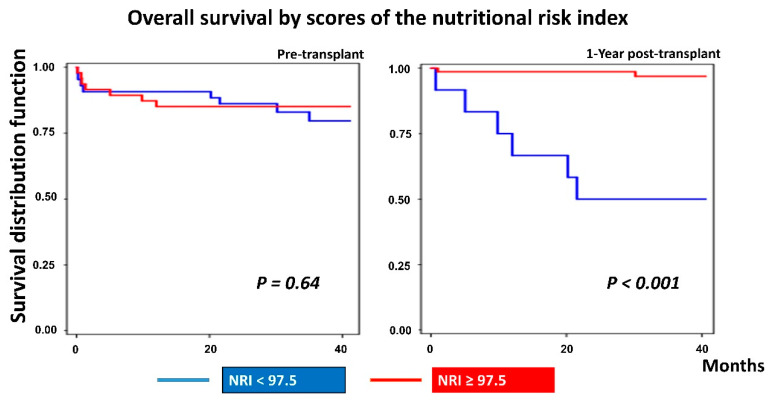
Kaplan–Meier plot of overall survival by NRI.

Kaplan–Meier survival curves for the preoperative and one-year postoperative NRI are shown in Figure 4. The NRI was classified into two groups: moderate-to-severe nutritional risk (NRI < 97.5) and mild-to-absent nutrition risk (NRI ≥ 97.5). Participants with moderate-to-severe nutrition risk had a lower survival by 81.40% (the mean survival time = 30.97 months) compared to the participants with mild-to-absent nutrition risk, who had a survival of 85.11% (the mean survival time = 10.84 months). However, interestingly, one year postoperative NRI showed significantly that mild-to-no nutritional risk patients had a higher percentage of living by 97.10% (the mean survival time = 29.77 months), compared to moderate-to-severe nutritional risk patients with 50.80% percentage of survival (the mean survival time = 16.5 months), (*p* < 0.001).

**Figure 5:**
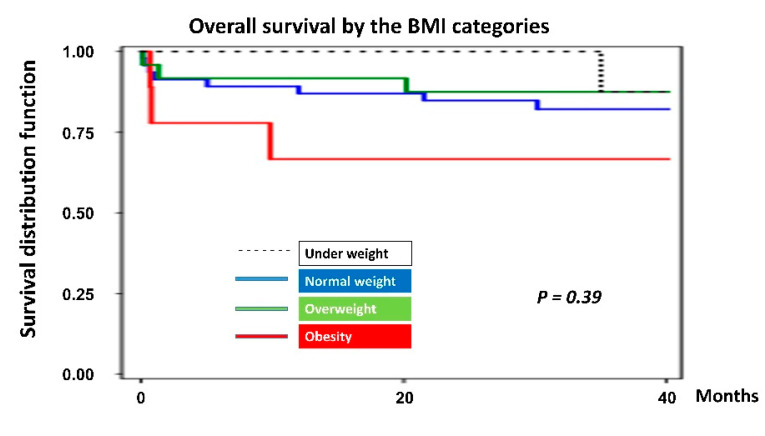
Kaplan–Meier graph of overall survival by body mass index categories.

Kaplan–Meier plot was also created for analysis of overall survival by BMI categories, from transplantation date until the time of death or the date of record of the last follow-up visit (Figure 5). Despite a lower survival of patients with obesity, there was no statistically significant difference among survival curves of all BMI categories (*p* = 0.39).

**Figure 6:**
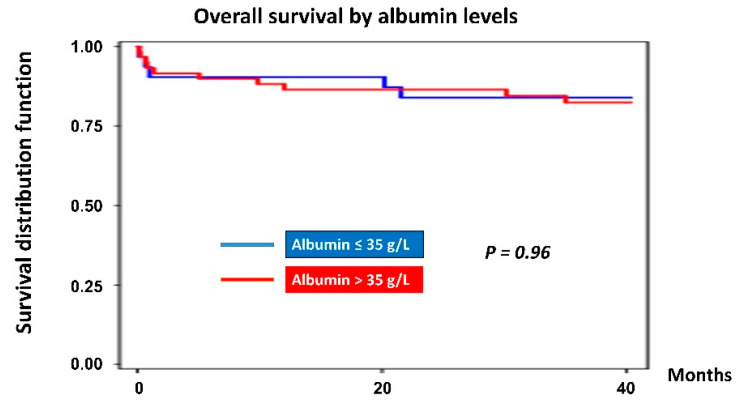
Kaplan–Meier graph of overall survival by albumin level.

Moreover, participants were divided into 2 groups based on pre-transplant albumin levels. According to nutritional status, the cutoff points used were >35 g/L (nourished group) and ≤35 g/L (malnourished group) [20]. The survival curves for both groups are demonstrated in Figure 6. There were no differences between the group with high pre-transplant albumin levels (the mean survival time = 30.67 months) compared with the group with low pre-transplant albumin levels (the mean survival time = 19.43 months) (83.05% vs. 83.87%, *p* = 0.96).

Table 4 summarizes the Cox proportional hazards regression analysis for some selected parameters. The most significant hazard ratio was postoperative NRI, which was 0.82 (95% CI, 0.75–0.89; *p* < 0.001). Recipients with higher BMI values were 1.5 times at risk of death than recipients with lower BMI values (HR = 1.535, 95% CI, 0–3669.1; *p* = 0.91).

## 4. Discussion

### 4.1. Main Findings

The present study was designed to evaluate the nutritional status among Saudi heart transplant patients who underwent transplantation by comparing NRI, anthropometric and biochemical measurements before and one year after heart transplantation and to demonstrate any nutritional-related gender difference.

Most of our recipients had DCM (52.22%) and ICM (32.22%). Similarly, a previous study found that the most underlying diseases were ICM (61%) and DCM (30%) among Spanish heart-transplanted patients [17]. This was also seen by Chou et al. (2006), who reported that the highest pre-transplant diagnoses were DCM (47%) and coronary artery disease (42%) in Chinese heart recipients [21].

Before heart transplantation, the prevalence of malnutrition risk (NRI < 100) was 60%. According to NRI, 7.78% were at severe nutritional risk; 40% were at moderate nutritional risk, and 12.22% were at mild nutritional risk. This result was similar to a Spanish study, which found, based on NRI, that 37% were at risk of malnutrition at the pre-transplant phase; where 5% were at severely nutritional risk, 22% were at moderate nutritional risk, and 10% were at mild nutritional risk [17]. The mean preoperative NRI was 96.60 ± 8.5, reflecting moderate nutritional risk, compared to their mean of 100.9 ± 9.9, reflecting no nutritional risk. Nonetheless, our participants were younger than their participants. The low scores of NRI in our population could be explained due to diverse healthcare systems and heart transplant protocols, long waiting periods until transplantation, and improper nutritional management during this period, resulting in the severe stage of malnutrition.

Moreover, other studies applied the NRI to a wide range of heart failure population. The reported prevalence of malnutrition risk ranges from 23% to 48%, and that of severe nutritional risk ranges from 2.8% to 15% [22,23,24]. Interestingly, though our patients had higher malnutrition risk, they were younger and had higher albumin levels than the others. Nevertheless, it is important to note that some calculated NRI by different equations. Some used the following formula: (1.5 × serum albumin, g/dL) + {41.7 × current weight (kg)/ideal weight (kg)} [23], while others used (1.5 × serum albumin, g/L) + {current weight (kg)/ideal weight (kg)} [22]. Finally, our population was transplant recipients, while they used heart failure patients.

Though a lot of nutritional assessment indices have been useful in anticipating the patient outcome, the best method to assess the nutritional status of hospitalized surgical patients is not yet established. Nonetheless, Kuzu and colleagues examined the best possible screening system in hospitalized surgical patients [25]. They applied some nutritional assessment tools to hospitalized surgical patients. These tools were the NRI, the Maastricht Index (MI), the Subjective Global Assessment (SGA), and the Mini Nutritional Assessment (MNA) for patients older than 59 years. They concluded that all these nutritional indices could be used safely in the clinical setting of surgical patients. Most importantly, they established a 63.5% prevalence of preoperative malnutrition using NRI. However, transplant patients are more compromised than any surgical patients because of complicated immunosuppressive regimens. The geriatric nutritional risk index, an updated version of NRI, was effective in predicting the length of hospital stay among older patients in non-cardiac surgical settings [26].

After heart transplantation, fortunately, the prevalence of nutritional risk decreased dramatically by 69.15% (from 60% to 18.51%), and the severe nutritional risk by 84% (from 7.78% to 1.23%) among our heart recipients. In other words, the mean preoperative NRI improved significantly from moderate nutritional risk to the absence of nutritional risk (96.60 ± 8.5 to 105.5 ± 8.1, *p* < 0.001). Simultaneously, BMI increased significantly after surgery. While the percentages of underweight and normal BMI categories decreased, the overweight and obesity prevalence increased. This could be attributed to the correction of heart failure symptoms after transplant and eventually improved nutritional status [10]. Also, steroids administration cause body composition modulation, change body metabolism and substantially induce weight gain [4,5,6].

It is important to recognize that the change of dietary habits is likely a key player in long-term metabolic abnormalities among patients who performed solid organ transplants [27]. Ferreira et al. did a nutritional and anthropometric evaluation for liver-transplanted patients for up to 12 months, where they linked post-transplant dietary changes and metabolic abnormalities [28]. A positive energy balance was noticed, and therefore an excessive weight (64%) was gained. They attributed it to an increased fat intake. Also, another study noticed the same thing of a high fat intake among post-renal-transplanted patients [29].

Moreover, this study showed that heart recipients with postoperative moderate or severe nutritional risk (NRI < 97.5) had significantly greater all-cause mortality in the first year post-transplantation than heart recipients with mild or no nutritional risk (NRI ≥ 97.5), as demonstrated in the Kaplan–Meier survival curve (*p* < 0.001). Additionally, participants with higher post-transplant NRI were 18% less likely to die than participants with lower post-transplant NRI (HR = 0.82; 95% CI, 0.75–0.89; *p* < 0.001). This was in line with Barge-Caballero et al., who observed a significant independent association between lower pre-transplant NRI and shorter post-transplant survival [17]. They showed that heart transplant recipients at mild or no nutritional risk (NRI ≥ 97.5) had a 45% lower risk of mortality in the first year after surgery than those with severe or moderate NRI (NRI < 97.5), (*p* = 0.001). Similarly, several studies showed the validity of NRI as an independent predictor of death and comorbidities among different stages of heart failure patients [22,23,24,30].

Besides, the Kaplan–Meier survival curve revealed that obese patients have a substantially shorter survival rate (*p* = 0.39). Moreover, obese patients were 1.5 times at risk of death than underweight patients (HR = 1.54; 95% CI, 0.00–3669.10; *p* = 0.91). These findings are following Russo and colleagues, who found a U-shaped relationship between the baseline BMI and the post-transplant survival [10]. They noticed a diminished survival among patients at the BMI extremes (underweight and obese II/III) compared with other groups. On the other hand, other studies reported an increased risk of mortality with lower BMI among cardiac surgical and heart failure patients [11,12,13,14,15,31]. This suggests that overweight and obesity may paradoxically have better outcomes than being underweight or normal among transplant recipients [15]. Nonetheless, a study showed that BMI did not predict six months’ mortality among advanced heart failure patients [30].

Furthermore, several studies have established a relationship between hypoalbuminemia and the morbidity and mortality of cardiovascular diseases [32]. Horwich et al. found that hypoalbuminemia heart failure patients were at higher risk of all-cause mortality and especially of heart failure mortality. Hypoalbuminemia can also help to select patients who required heart transplantation urgently [33]. Nonetheless, in contrast to their findings, in our investigation, there were no differences between the group with high pre-transplant albumin levels compared with the group with low pre-transplant albumin levels (83.05% vs. 83.87%, *p* = 0.96). Likely, Rapp-Kesek et al. (2004), showed no association between albumin and mortality among cardiac surgery patients. However, albumin was associated with infection [13]. Nevertheless, the mean survival time of the hypoalbuminemia group was lower than the normoalbuminuric group (19.43 months vs. 30.67 months).

The worthy observation in this study regarding gender differences in the small percentage of female heart transplant candidates (only about 22%) in comparison to males. Likely, other studies reported a significantly higher proportion of males among heart recipients 69.2% and 83%, respectively [17,34]. Additionally, Stein et al. found that the majority of their heart and liver transplanted patients were males (81%) [35]. This was also reported in the field of liver transplantation, as the reports suggest that women are also less likely to undergo liver transplantation once listed and consequently have greater morbidity or even mortality compared to men [36]. Furthermore, the same inequity is apparent in kidney transplantation. This gender inequity could be due to the greater economic status of men, lack of social support, and different health-seeking behaviors of women [37].

Before heart transplantation, women were significantly less in body weight than men with an insignificant difference in NRI scores, while after one year of the surgery, the NRI score was significantly different in favor of men. Survival analysis showed an insignificant difference in mortality among men and women; however, Dang et al. [38] reported that female sex was an independent predictor of the early mortality after the use of left ventricular assist devices for patients with uncompensated congestive heart failure. On the contrary Almufleh et al. [39] reported that survival or cardiac graft function showed no gender difference. However, women had higher rates of infectious complications than men and more frequent re-hospitalization for infection. In a recent long-term study over 25 years, the mortality rate was higher in women due to the rejection of heart transplants and primary heart failure [40].

### 4.2. Strengths and Limitations

The present study had several strengths. The appropriate sample size was sufficiently representative of this disease population in our locality. We also used a validated assessment tool (NRI) in addition to anthropometric and biochemical domains of nutritional assessment. The survival analysis was also done to prove the importance of nutritional support and the impact of malnutrition on the survival of heart transplant patients.

The present study had some limitations, as this work was retrospective, and we recommend validation of our results in a prospective study. Regarding the calculation of the NRI, we used the recorded usual body weight in patients’ files to calculate the IBW, then the IBW was used in the modified NRI formula according to Barge-Caballero et al. [17]. Despite the validity and high sensitivity of the NRI, it encounters some limitations as it depends on body weight measurements which may be affected by changes in the total body water, and also depend on albumin level which has some non-nutritional modulating factors [41]. Furthermore, we did not compare NRI with other nutritional assessment tools.

## 5. Conclusions

The field of organ transplantation continues to increase as the number of transplants performed rises each year, new medical advances are developed, and the survival rate of candidates improves. Unfortunately, because of a critical scarcity of available hearts for transplantation, achieving maximal benefit from this therapy is required through improving modifiable risk factors such as nutritional status. In this study, the prevalence of nutritional risk was high among Saudi heart transplants before transplantation. Special attention should be paid early to this population during this period.

After transplantation, nutritional status improved significantly. The NRI after 1 year of transplant correlated significantly with mortality. Generally, females were at higher nutritional risk than males. Hence, further intensive nutritional management is needed for this specific population. Given the high prevalence of nutritional risk in heart transplanted patients, the establishment of strategies aimed to optimize the nutritional status is urgently needed to reduce morbidity and mortality.

## Figures and Tables

**Table 1:** Gender difference in baseline general characteristics.

Variables	Males (*n* = 70) Mean ± SD	Females (*n* = 20) Mean ± SD	*p*-Value
Length of stay	28.81 ± 27.06	28.75 ± 23.97	NS
Age	39.84 ± 12.22	32.35 ± 9.31	<0.05
Height (cm)	169.30 ± 6.68	157.60 ± 4.31	<0.001
Ideal weight (kg)	65.21 ± 6.01	52.35 ± 2.80	<0.001
Weight (kg)	70.19 ± 15.44	55.43 ± 13.50	<0.001
Body mass index (kg/m^2^)	24.48 ± 5.06	22.20 ± 5.02	NS
Nutritional risk index	96.97 ± 8.52	95.29 ± 8.56	NS

NS; Nonsignificant *p*-value (*p*-value > 0.05).

**Table 2:** Study variables measurements pre and post-transplantation.

Variables	Pre-Transplant (*n* = 90)		Post-Transplant (*n* = 90)		*p*-Value
	Range	Mean ± SD	Range	Mean ± SD	
Weight (kg)	32.70–117.00	66.91 ± 16.18	31.00–139.40	73.50 ± 17.88	<0.001
BMI (kg/m^2^)	13.27–39.09	23.98 ± 5.11	12.74–43.02	26.40 ± 5.68	<0.001
NRI	69.10–114.60	96.60 ± 8.50	72.10–120.70	105.50 ± 8.10	<0.001
Albumin (g/L)	23.00–48.00	37.61 ± 5.23	20.00–52.00	41.99 ± 5.86	<0.001

NRI: Nutritional Risk Index.

**Table 3:** Study variables measurements pre- and post-transplantation according to gender.

Variables	Pre-Transplant		*p*-Value	Post-Transplant		*p*-Value
	Male (*n* = 70) Mean ± SD	Female (*n* = 20) Mean ± SD		Male (*n* = 70) Mean ± SD	Female (*n* = 20) Mean ± SD	
Hemoglobin (g/L)	119.30 ± 22.91	105.00 ± 18.52	<0.05	129.80 ± 23.05	105.90 ± 17.76	<0.001
Hematocrit (%)	36.00 ± 7.00	32.00 ± 5.00	<0.05	39.00 ± 7.00	33.00 ± 5.00	<0.001
Lymphocyte count (%)	18.74 ± 15.98	23.28 ± 15.53	NS	20.29 ± 11.17	22.49 ± 14.27	NS
Red blood cells (1012/L)	4.52 ± 00.95	3.97 ± 0.69	<0.05	4.85 ± 00.92	3.89 ± 0.65	<0.001
Iron (µmol/L)	11.53 ± 6.43	12.67 ± 14.14	NS	12.02 ± 5.28	8.86 ± 5.46	NS
TIBC (µmol/L)	63.29 ± 12.09	61.46 ± 13.21	NS	45.20 ± 10.73	46.90 ± 9.18	NS
UIBC (µmol/L)	51.55 ± 12.76	50.11 ± 16.18	NS	33.31 ± 12.39	37.94 ± 10.39	NS
Ferritin (µg/L)	183.80 ± 183.20	145.60 ± 178.20	NS	379.50 ± 331.10	323.90 ± 407.00	NS
Alkaline phosphatase (u/L)	111.90 ± 72.35	102.30 ± 69.03	NS	104.10 ± 70.89	110.00 ± 80.33	NS
Total protein (g/dL)	72.72 ± 11.49	68.17 ± 11.86	NS	70.39 ± 7.96	64.54 ± 7.93	<0.05
Albumin (g/L)	37.72 ± 5.18	37.20 ± 5.53	NS	42.52 ± 6.02	39.05 ± 4.93	NS
Prealbumin (mg/dL)	187.80 ± 59.36	116.30 ± 56.07	<0.05	293.10 ± 68.01	167.80 ± 108.10	<0.05
Total cholesterol (mmol/L)	2.98 ± 1.06	3.08 ± 1.02	NS	3.72 ± 1.49	3.80 ± 0.79	NS
Low Density Lipoprotein (mmol/L)	1.98 ± 0.76	2.05 ± 0.69	NS	2.23 ± 1.06	1.96 ± 0.49	NS
High Density Lipoprotein (mmol/L)	0.88 ± 0.38	0.99 ± 0.41	NS	1.20 ± 0.39	1.46 ± 0.49	NS
Triglycerides (mmol/L)	0.85 ± 0.36	0.86 ± 0.43	NS	1.35 ± 0.60	1.32 ± 0.63	NS
Triiodothyronine (mmol/L)	1.35 ± 0.46	1.35 ± 0.39	NS	1.61 ± 0.41	1.24 ± 0.65	NS
Free thyroxine (pmol/L)	20.09 ± 5.22	19.81 ± 5.58	NS	17.80 ± 4.22	18.01 ± 5.66	NS
Thyroid stimulating hormone (mU/L)	4.71 ± 3.90	4.53 ± 2.72	NS	3.01 ± 2.50	2.45 ± 2.21	NS
Fasting blood glucose (mmol/L)	6.23 ± 2.85	6.73 ± 3.85	NS	6.47 ± 2.73	6.61 ± 3.92	NS
HbA1C (%)	6.90 ± 2.01	6.09 ± 1.71	NS	6.87 ± 1.80	6.18 ± 2.12	NS

TIBC; total iron-binding capacity, UICB; unsaturated iron-binding capacity, HbA1c; glycosylated hemoglobin. NS; Nonsignificant *p*-value (*p*-value > 0.05).

**Table 4:** Cox proportional hazards regression analysis of some characteristics of survival.

Variables	Hazard Ratio	95% CI	*p*-Value
Pre-transplant NRI	0.79	0.92–1.02	0.20
Post-transplant NRI	0.82	0.75–0.89	<0.001
BMI	1.54	0–3669.1	0.91
Lymphocytes	0.98	0.83–1.15	0.78
Total cholesterol	0.79	0.02–31.79	0.91
